# Insights into the metabolic profiling of Polygonati Rhizoma fermented by *Lactiplantibacillus plantarum* under aerobic and anaerobic conditions using a UHPLC-QE-MS/MS system

**DOI:** 10.3389/fnut.2023.1093761

**Published:** 2023-01-26

**Authors:** ZiLing Wang, Jia Lao, XingYi Kang, ZhenNi Xie, Wei He, XiaoLiu Liu, Can Zhong, ShuiHan Zhang, Jian Jin

**Affiliations:** ^1^Hunan Academy of Chinese Medicine, Hunan University of Chinese Medicine, Changsha, Hunan, China; ^2^Institute of Chinese Medicine Resources, Hunan Academy of Chinese Medicine, Changsha, Hunan, China; ^3^Resgreen Group International Inc., Changsha, China; ^4^College of Mechanical and Energy Engineering, Shaoyang University, Shaoyang, Hunan, China

**Keywords:** fermentation, Polygonati Rhizoma, *Lactiplantibacillus plantarum*, metabolomics, aerobic, anaerobic

## Abstract

**Introduction:**

Polygonati Rhizoma is a multi-purpose food with medicinal uses. Fermentation of Polygonati Rhizoma by lactic acid bacteria could provide new insights into the development of Polygonati Rhizoma products.

**Methods:**

In this study, *Lactiplantibacillus plantarum* was fermented with Polygonati Rhizoma extracts in a bioreactor under aerobic and anaerobic conditions with pH and DO real-time detection. Metabolic profiling was determined by UHPLC-QE-MS/MS system. Principal component analysis and orthogonal partial least-squares discriminant analysis were used to perform multivariate analysis.

**Results:**

A total of 98 differential metabolites were identified in broth after fermentation, and 36 were identified between fermentation under aerobic and anaerobic conditions. The main metabolic pathways in the fermentation process are ABC transport and amino acid biosynthesis. Most of the compounds such as L-arginine, L-aspartic acid, leucine, L-lysine, citrate, inosine, carnitine, betaine, and thiamine were significantly increased during fermentation, playing a role in enhancing food flavor. Compared with anaerobic fermentation, aerobic conditions led to a significant rise in the levels of some compounds such as valine, isoleucine, and glutamate; this increase was mainly related to branched-chain amino acid transaminase, isocitrate dehydrogenase, and glutamate dehydrogenase.

**Discussion:**

Aerobic fermentation is more beneficial for the fermentation of Polygonati Rhizoma by L. *plantarum* to produce flavor and functional substances. This study is the first report on the fermentation of Polygonati Rhizoma by L. *plantarum* and provides insights that would be applicable in the development of Polygonati Rhizoma fermented products.

## 1. Introduction

Polygonati Rhizoma is the dried rhizome of *Polygonatum kingianum* Coll. et Hemsl, *Polygonatum sibiricum* Red., or *Polygonatum cyrtonema* Hua (1). It contains a variety of active ingredients, such as polysaccharides, steroidal saponins, triterpenes, alkaloids, lignans, flavonoids, phytosterols, and volatile oils (2). As a multi-purpose medicinal and edible homologous resource, the value of Polygonati Rhizoma is not only reflected in its pharmacological effects but also has great potential as an important food source, because it does not require fertile soil to grow (3). Generally, the fresh Polygonati Rhizoma is not being eaten directly, but with processing treatment. For instance, it is usually processed with several times steaming and drying. Recently, fermentation technology is also applied in the processing of Polygonati Rhizoma. Fermented Polygonati Rhizoma has been developed into healthy food, and moreover, Polygonati Rhizoma with fermentation is also proposed to be used as a beneficial product related to agricultural organic fertilizer and animal breeding industry (4).

Lactic acid bacteria are a type of probiotic, which can colonize the human body and provide benefits to the host by altering the composition of the microbiota of a certain body part (5). With an in-depth understanding of lactic acid bacteria, various active ingredients and uses of lactic acid bacteria can be made known for the benefit of the general public. For example, it has the potential to detoxify contaminated food, reduce the toxicity of patulin (6), and display an inhibitory effect on Coxsackie virus B4 (7). The fermentation of lactic acid bacteria is a particularly complex process resulting from the combination of several factors, such as temperature, humidity, and oxygen content. Studies have shown that the same bacteria may behave differently under different oxygen conditions because most lactic acid bacteria are facultative anaerobes. For instance, substantial differences were observed in catabolic end-products of glucose between aerated and anaerobic cultures of *Lactiplantibacillus plantarum* (8).

In addition to lactic acid bacteria, other organisms, e.g., yeasts and fungi are commonly used as microbial starters for accelerating desirable fermentation processes and decreasing the risk of fermentation failure. Some studies on the effects of different microbial starters have provided fundamental knowledge for improving the quality of fermented Polygonati Rhizoma products. However, previous studies have mainly focused on microbial starter yeast (9) and *Monascus purpureus* (10). Up to now, there are few research studies on the effects of lactic acid bacteria in Polygonati Rhizoma fermentation.

In this study, the Polygonati Rhizoma extracts were fermented by the typical lactic acid bacterium, *L. plantarum*, which was included in the issued list of bacteria that can be used in food by the Chinese Ministry of Health in 2010 and regarded as a safe species for food development (11). The fermentation processes were carried out under aerobic and anaerobic conditions in a bioreactor system. The changes in pH and dissolved oxygen (DO) in the fermentation broths of Polygonati Rhizoma were monitored in real-time. Metabolic profiling and metabolomics analysis were conducted using UHPLC-QE-MS/MS. To the best of our knowledge, this study is the first time to investigate the fermentation of Polygonati Rhizoma by lactic acid bacteria in a well-controlled bioreactor system under different fermentation environments, and it could provide insights for further research and development of Polygonati Rhizoma products with using fermentation broth as a source of healthy molecules.

## 2. Materials and methods

### 2.1. Polygonati Rhizoma and extraction

The Polygonati Rhizoma used for fermentation was produced by Yipuyuan Inc., China. Firstly, 20 g of Polygonati Rhizoma was extracted with 1 L water at 100°C for 1 h. Then, the extracts were filtered with gauze, and the residual Polygonati Rhizoma was extracted once again. Both extracts were merged and used for further fermentation.

### 2.2. Strain and preculture

The *L. plantarum* microbial starter was procured from Shandong Zhongke-Jiayi Biological Engineering Co., Ltd., China. It was cultured in an MRS medium, in which 1 L medium contained 10 g of peptone, 10 g of yeast extract, 20 g of dextrose, 2 g of ammonium citrate, 5 g of sodium acetate, 0.5 g of MgSO_4_⋅7H_2_O, and 0.5 g of tween(R) 80. The strain was cultivated on a shaker at 37°C for 16 h before inoculation. The inoculum was cultivated on a shaker at 37°C for 16 h, and the strain was harvested for inoculation.

### 2.3. Bioreactor system

**FIGURE 1:**
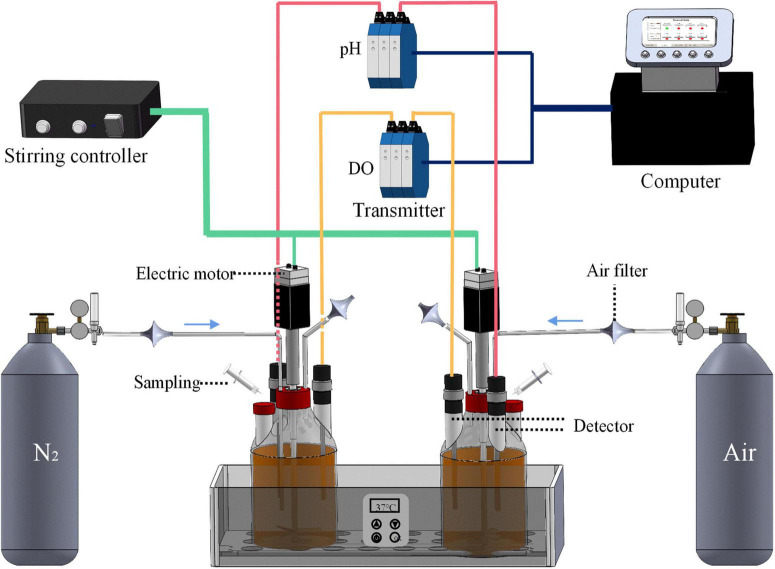
The bioreactor system used for the fermentation of Polygonati Rhizoma with *Lactiplantibacillus plantarum* under aerobic and anaerobic conditions. The pH detector and DO detector are connected to the computer through the transmitter, which can monitor the values of pH and DO of the broths in real-time, and promptly feedback the results to the computer.

A bioreactor system with independent 2 L fermentation bottles obtained from Bipu Huarui Scientific Instruments (Beijing) Co., Ltd., China, was used for fermentation, as shown in Figure 1. Fermentation bottles were autoclaved *via* a vertical high-pressure steam sterilizer (Shanghai Shenan Medical Instrument Factory, China). The pH and DO detectors were sterilized by a vapor stream generated by a vapor generator (Shanghai Baisheng Washing Equipment Co., Ltd., China). The temperature was regulated at 37 ± 1°C by thermostatic control. The speed of the axial flow impellers was 100 rpm. The gas inlet was connected to either the air bottle or nitrogen gas bottle to create an aerobic or anaerobic condition, with a gas flow rate set at 2.5 mL/min. The pH and DO values in fermentation broths were detected in real-time through the pH and DO detectors, which were monitored and recorded by the computer. Apart from the online real-time detection, a hand-held offline detector (Knick Elektronische Messgeräte GmbH & Co., Germany) was also used to calibrate the pH and DO data.

### 2.4. Fermentation process

Each fermentation bottle was filled to its 2 L working volume with extracts from 20 g of Polygonati Rhizoma. Next, *L. plantarum* at a concentration of about 5 × 10^9^ CFU/mL was inoculated into the fermentation bottles to start the fermentation process. Then, three bottles were connected to the air supply to create aerobic conditions, and the other three bottles had their gas inlets connected to pure nitrogen gas to create anaerobic conditions *via* a continuous supply of gas into the fermentation bottles. The growth of the *L. plantarum* was counted every day referred to the method described before (12), and the specific growth rate (μ) was calculated as the equation (1) (13) as following.


(1) $$\mu =\frac{1}{X}\,\frac{d\,X}{d\,t}$$

After 7 days of fermentation, six samples from each group were centrifuged immediately, and the centrifugal precipitation cells and supernatants were used for UHPLC-MS/MS analysis. The samples were named AC, aerobic fermentation *L. plantarum* cells; NC, anaerobic fermentation *L. plantarum* cells; AS, aerobic fermentation supernatants; NS, anaerobic fermentation supernatants; and EB, extractives of Polygonati Rhizoma before fermentation.

### 2.5. UHPLC-MS/MS analysis

Metabolomics profiling was performed using a UHPLC-MS/MS system (UHPLC, Shimadzu Nexera X2 LC-30AD, Shimadzu, Japan) in combination with Q-Exactive Plus (Thermo Scientific, San Jose, USA). For hydrophilic interaction liquid chromatography separation, the samples were analyzed using a 2.1 mm*100 mm ACQUIY UHPLC BEH Amide 1.7 μm column (Waters, Ireland). The flow rate was 0.5 mL/min, and the mobile phase contained A: 25 mM ammonium acetate and 25 mM ammonium hydroxide in water and B: 100% acetonitrile (ACN). The gradient was kept at 95% B for 1 min and linearly reduced to 65% in 7 min. Then, it was reduced to 35% in 2 min and maintained for 1 min, before being increased to 95% in 0.5 min with a 2 min re-equilibration period employed. Both positive-mode and negative-mode electrospray ionization (ESI) were applied for MS data acquisition. The ESI source conditions were set as follows: spray voltage of 3.8 kv (+) and 3.2 kv (−); capillary temperature of 320 (±); sheath gas of 30 (±); aux gas of 5 (±); probe heater temp of 350 (±); and S-Lens RF level of 50. In the MS-only acquisition, the instrument was programmed to acquire over the m/z range of 80–1,200 Da. The full MS scans were acquired at resolutions of 70,000 at m/z 200 and 17,500 at m/z 200 for the MS/MS scan. The maximum injection times were set at 100 ms for MS and 50 ms for the MS/MS scan. The isolation window for MS2 was set to 2 m/z, and the normalized collision energy (stepped) was set as 27, 29, and 32 for fragmentation. Quality control (QC) samples were prepared by pooling aliquots of all samples that were representative of the samples under analysis, and these QC samples were used for data normalization. Blank samples (75% ACN in water) and QC samples were injected every six samples during acquisition.

### 2.6. Preprocessing and filtering of data

The raw MS data were processed using MS-DIAL for peak alignment, retention time correction, and peak area extraction, and then compared with databases, which include public MS/MS libraries MassBank, NIST14, ReSpect, HMDB, etc. These databases were converted in MSP format and merged with MoNA-MassBank of North America database, and a self-built metabolite standard library generated by Shanghai Bioprofle Biological Technology Co., Ltd. In the extracted-ion features, only variables with more than 50% of the non-zero measurement values in at least one group were kept.

### 2.7. Multivariate statistical analysis

The software R (version 4.0.3) with packages was used for all multivariate data analyses and modeling. The data were mean-centered using Pareto scaling, and the models were built on PCA and OPLS-DA. All evaluated models were tested for overfitting using the permutation test method. Mathematically, the scores were calculated for each variable as a weighted sum of squares of PLS weights. The mean VIP value was 1, and VIP values over 1 were usually considered to be significant. The discriminating metabolites were obtained using a statistically significant threshold of VIP values obtained from the OPLS-DA model and a two-tailed Student’s *t*-test (*p*-value) on the normalized raw data at the univariate analysis level. The one-way ANOVA test (one-way variance test) was used to compare the metabolites in pairs to analyze the significance of the metabolites, and then the metabolites with *p*-value < 0.05 and OPLS-DA VIP > 1 were screened as statistically significant metabolites. Fold change was calculated as the logarithm of the average mass response ratio between two arbitrary classes.

### 2.8. KEGG enrichment analysis

To identify biological pathways, the differential metabolite data were analyzed using KEGG pathway enrichment analysis against the KEGG database. KEGG enrichment analysis was carried out with Fisher’s exact test, and FDR correction for multiple testing was performed. The enriched KEGG pathways were nominally statistically significant at the *p* < 0.05 level.

## 3. Results and discussion

### 3.1. Real-time detection of pH, DO, and cells growth

The fermentation process of Polygonati Rhizoma is a complex process involving dynamic changes in microorganisms, enzymes, and chemical components. *L. plantarum* is a facultative anaerobic bacterium, and it can grow under both aerobic and anaerobic conditions (8). The pH and DO are important basic indicators that can be used to measure changes in the fermentation process. In this experiment, a bioreactor was used to monitor the Polygonati Rhizoma fermentation process in real-time.

**FIGURE 2:**
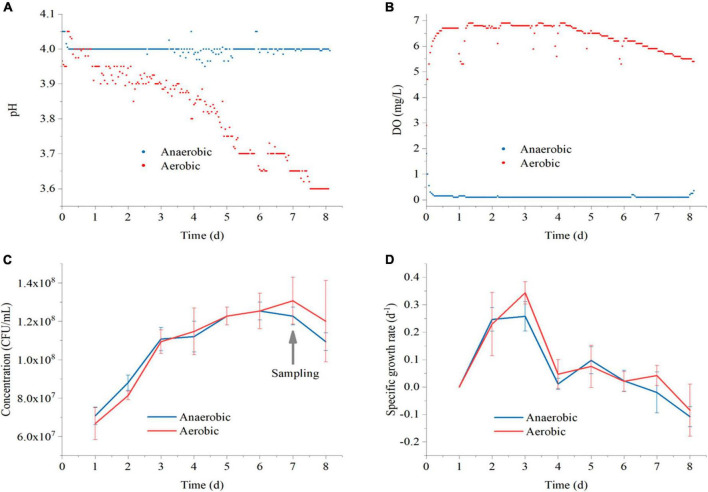
The real-time values of pH **(A)** and DO **(B)** in Polygonati Rhizoma broths fermented by *Lactiplantibacillus plantarum* under aerobic and anaerobic conditions and the concentration of cells **(C)** and average specific growth **(D)** in Polygonati Rhizoma broths fermented by *L. plantarum* under aerobic and anaerobic conditions. DO, dissolved oxygen.

As shown in Figures 2A, B, the pH in anaerobic conditions was relatively stable at a value of 4.0 ± 0.1. In aerobic conditions, the pH decreased gradually from 4.0 to 3.6, indicating that more organic acid metabolites were produced in the aerobic environment. The DO was stable at about 0 mg/L in the anaerobic environment and 6.0 mg/L in the aerobic environment, confirming that the anaerobic and aerobic environments were well maintained during the experiment.

The concentration of *L. plantarum* (C) and its average specific growth (D) in Polygonati Rhizoma broths under aerobic and anaerobic conditions are show in Figure 2. In the aerobic and anaerobic environment, the concentration of cells increased continuously from days 1 to 7, achieved the maximum on day 7, and began to decline on day 8. The average specific growth rate of *L. plantarum* increased rapidly during the 1st to 3rd day, reached a maximum value on the 3rd day. Then, the specific growth rate decreased to about 0 d^–1^ on day 7 and changed to negative growth on the 8th day. Therefore, we took the fermentation samples for metabolomics analysis on 7th day, since the concentration of *L. plantarum* was achieved highest and the fermentation was already complete at this stage.

### 3.2. Principal component analysis (PCA)

Among the multivariate techniques, PCA is a well-known unsupervised technique, and the initial step of multivariate analysis was used to outline overall data with dimension reduction (14).

**FIGURE 3:**
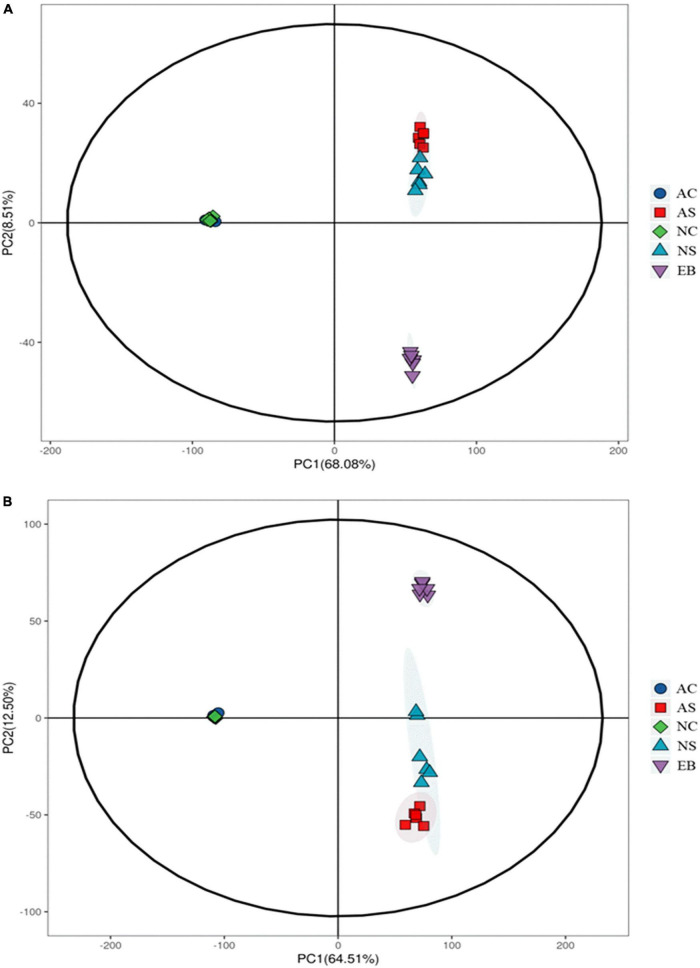
PCA score plots of positive **(A)** and negative **(B)** metabolite profiling data of Polygonati Rhizoma fermented using *Lactiplantibacillus plantarum* under aerobic and anaerobic conditions. AC, aerobic fermentation with *L. plantarum* cells; NC, anaerobic fermentation with *L. plantarum* cells; AS, aerobic fermentation supernatants; NS, anaerobic fermentation supernatants; EB, extracts of Polygonati Rhizoma before fermentation.

As shown in Figure 3, PCA of the metabolomics data of the positive mode produced two PCs that explained more than 76.59% in the total variance (PC1 and PC2 explained 68.08 and 8.51%, respectively). At the same time, the PCA of the metabolomics data of the negative mode produced two PCs that explained more than 77.01% of the total variance (PC1 and PC2 explained 64.51 and 12.50%, respectively). The *L. plantarum* cell groups under aerobic and anaerobic conditions were overlapping for both the positive and negative metabolite profiling data, indicating that no obvious difference existed between these two cell groups. Interestingly, the three groups of broth supernatants, i.e., the extracts before fermentation, the broths fermented under aerobic conditions, and the broths fermented under anaerobic conditions, were well separated along PC2, reflecting differences in their metabolic profiles (15). All the samples in the score plot were within 95% of the Hotelling’s T2 ellipse and the QC clustered together (Supplementary Figure 1), showing the good reproducibility and stability of the employed metabolomics method (16).

### 3.3. Orthogonal partial least-squares discriminant analysis (OPLS)

Even though unsupervised analyses, such as PCA, are useful for investigating overall sample patterns, they do not allow one to easily identify common properties for each variation. On the other hand, OPLS-DA is a suitable tool for reducing non-correlated variation and model complexity with improved interpretational ability. Not only that, using it appropriately can often yield beneficial insights into common and predictive features in each sample group (17).

**FIGURE 4:**
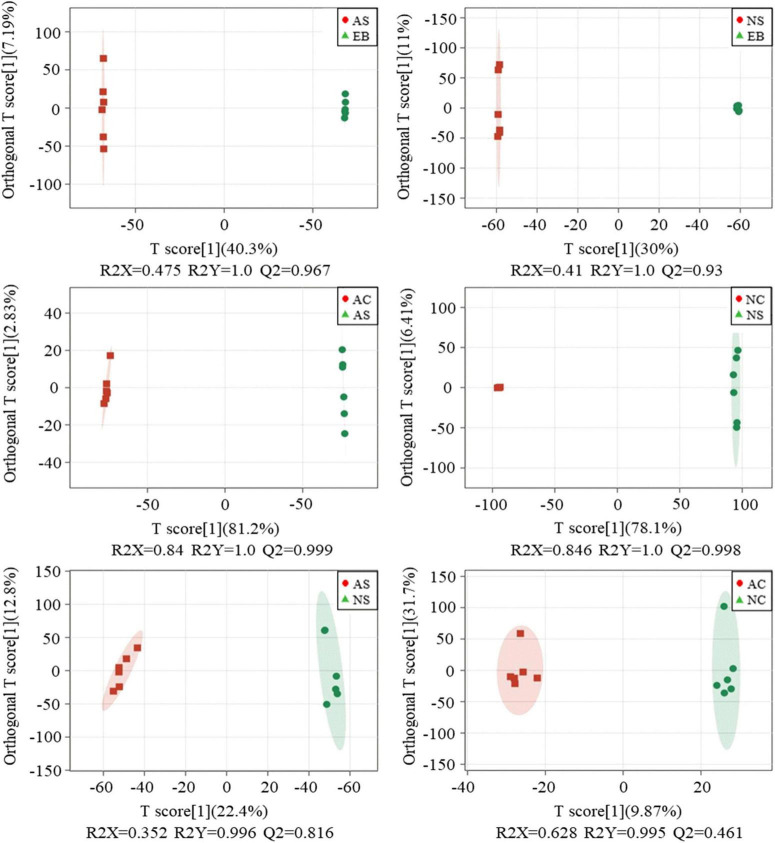
OPLS-DA analysis of profiling data of Polygonati Rhizoma fermented using *Lactiplantibacillus plantarum* under aerobic and anaerobic conditions. AC, aerobic fermentation *L. plantarum* cells; NC, anaerobic fermentation *L. plantarum* cells; AS, aerobic fermentation supernatants; NS, anaerobic fermentation supernatants; EB, extractives of Polygonati Rhizoma before fermentation.

To maximize the separation within groups, OPLS-DA was used to compare the metabolomics data between the six approaches for pairwise comparisons. The permutation test was used to verify and evaluate the quality of the OPLS-DA model. OPLS-DA score plots consisting of a sample with different treatments are shown in Figure 4, and a clear separation between groups can be seen. The high values of R2 (close to 1) and Q2 (> 0.8) values for each OPLS-DA model except for the AC vs. NC group indicate that the models were stable and appropriate for fitness and prediction. Additionally, these data confirmed that the extracts of Polygonati Rhizoma fermented under aerobic and anaerobic conditions contained different ingredients. The specific different components will be further selected by significance analysis.

### 3.4. Differential metabolite selection

**FIGURE 5:**
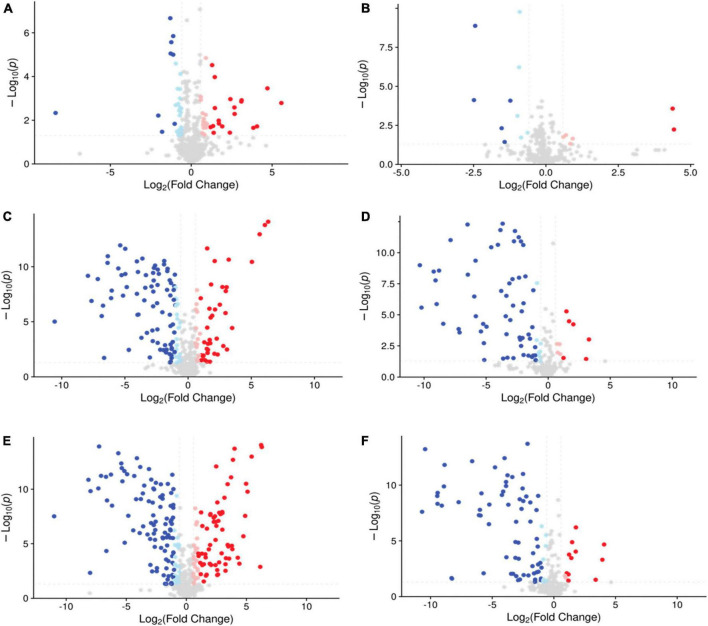
The volcano plot of the comparison group AS vs. NS in negative mode **(A)** and positive mode **(B)**; EB vs. AS in negative mode **(C)** and positive mode **(D)**; EB vs. NS in negative mode **(E)** and positive mode **(F)**, with FC > 1.5 or FC < 0.667, and *P*-value < 0.05 as the screening criteria, the vertical dotted line analysis indicates log2(1/1.5) and log2(1.5); the blue point means Log2(FC) <−1 and the light blue point means −1 < Log2(FC) < 0, both of them indicating down-regulated metabolites; the pink point means 0 < Log2(FC) < 1 and the red point means Log2(FC) > 1, both of them indicating up-regulated metabolites; AS, aerobic fermentation supernatants; NS, anaerobic fermentation supernatants; EB, extractives of Polygonati Rhizoma before fermentation.

To obtain a clearer view of compound metabolism, volcano plots were used to show pairwise comparisons of metabolomic data. Volcano plots composed of samples with different treatments are shown in Figure 5, presenting abundant differential metabolites between the two groups. The group of AS vs. NS had 151 differential metabolites in the negative mode and 16 differential metabolites in the positive mode. The comparison of EB vs. AS revealed 210 differential metabolites in the negative mode and 87 differential metabolites in the positive mode. Additionally, there were 216 differential metabolites in the negative mode and 77 differential metabolites in the positive mode in EB vs. NS. These data confirm that the Polygonati Rhizoma broths fermented under aerobic conditions, anaerobic conditions, and extracts without fermentation had different compositions. The specific different components will be further selected by KEGG analysis.

### 3.5. Classification and KEGG enrichment

**FIGURE 6:**
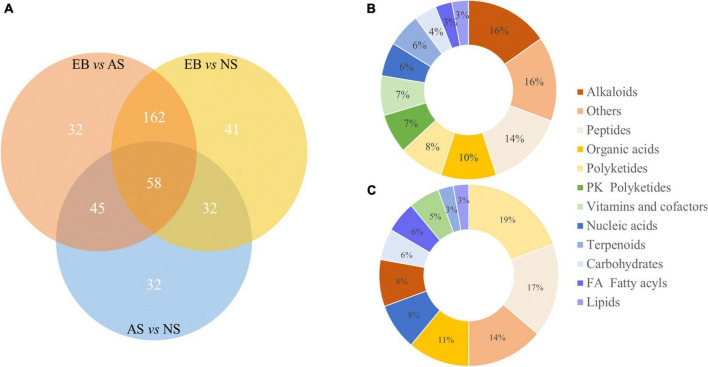
Venn diagram of differential metabolites in the three comparison groups of EB vs. AS, EN vs. NS, and AS vs. NS **(A)**, and KEGG classification of significant different metabolites before and after fermentation **(B)** and under aerobic and anaerobic conditions **(C)**. AS, aerobic fermentation supernatants; NS, anaerobic fermentation supernatants; EB, extractives of Polygonati Rhizoma before fermentation.

There were 297 significantly different metabolites in EB vs. AS group (Supplementary Table 1), 293 in EB vs. NS group (Supplementary Table 2), and 167 in AS vs. NS group (Supplementary Table 3). They were further divided into two comparison groups that the group of before fermentation and after fermentation (EB vs. AS and NS), and group of fermentation in aerobic and anaerobic environment (AS vs. NS). The significantly different metabolites in these two groups were classified by KEGG categorization. A total of 98 of metabolites were identified by database matching in EB vs. AS and NS groups, and 36 in AS vs. NS group. In the EB vs. AS and NS group, 15 alkaloids (16%), 14 peptides (14%), 10 organic acids (10%), and other differential metabolites were included. The AS vs. NS contained seven PK Polyketides (19%), six Peptides (17%), and other differential metabolites, as shown in Figure 6. KEGG classification results showed that organic acids and their derivatives accounted for a high proportion in the two comparison groups, which was related to the addition of *L. plantarum* to the fermentation broth. Lactic acid bacteria are characteristically known to produce organic acids (5). In a previous study, *L. plantarum* was reported to produce high concentrations of lactic and acetic acids in orange juice (18).

**FIGURE 7:**
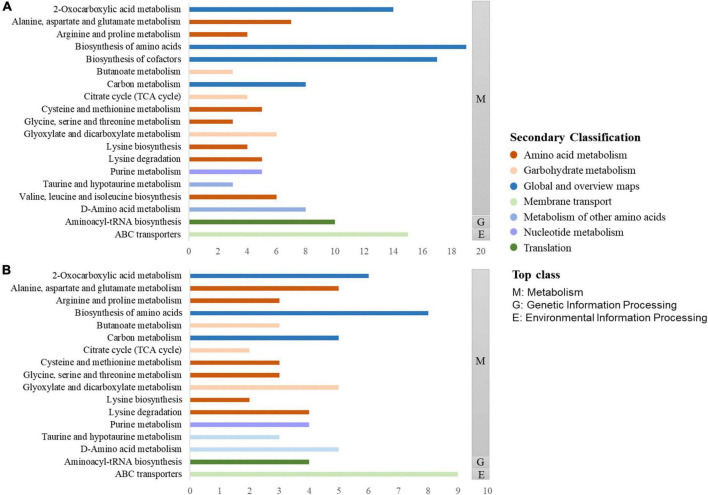
KEGG enrichment pathways of differential metabolites in Polygonati Rhizoma fermented by *Lactiplantibacillus plantarum* before and after fermentation **(A)** and under aerobic and anaerobic conditions **(B)**.

The KEGG database is typically used to explore metabolic pathways and elucidate the mechanisms of metabolic changes during fermentation. As displayed in Figure 7, the differential metabolites of the two groups can be divided into three top-class: metabolism, genetic information processing, and environmental information processing. Metabolism mainly includes biosynthesis of amino acid, 2-oxocaboxylic acids metabolism, and other pathways. Genetic information processing included aminoacyl-tRNA biosynthesis, and environmental information processing included ABC transporters. Combined with Figures 7A, B, it is found that the two most enriched pathways were ABC transporters and biosynthesis of amino acids. As a large family of ATP-dependent transmembrane proteins, ABC transporters have an important impact on the *in vivo* behavior of most of the currently used drugs, including drugs for the treatment of tumors, AIDS, and microbial infections (19). Amino acids are the building blocks that support life. They have well-defined roles in protein synthesis and contribute to many other intracellular metabolic pathways, including ATP production, nucleotide synthesis, and redox balance, to support cellular and organismal functions (20).

### 3.6. Main metabolites produced by fermentation

**FIGURE 8:**
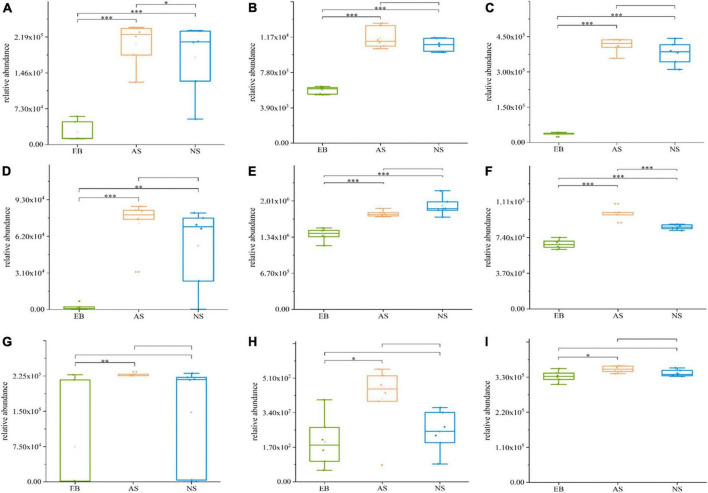
Boxplots of partially differential metabolites enriched in ABC transporters and biosynthesis of amino acid pathways in Polygonati Rhizoma fermented by *Lactiplantibacillus plantarum* under aerobic and anaerobic conditions. **(A)** L-arginine; **(B)** L-aspartic acid; **(C)** leucine; **(D)** L-lysine; **(E)** citrate; **(F)** inosine; **(G)** carnitine; **(H)** betaine; and **(I)** thiamine. AS, aerobic fermentation supernatants; NS, anaerobic fermentation supernatants; EB, Polygonati Rhizoma extractives before fermentation. *p < 0.05, **p < 0.01, and ***p < 0.001.

In the EB vs. AS and NS groups, a total of 19 differential metabolites were transported by ABC transporters, and most of the compounds enriched in ABC transporters and biosynthesis of amino acid pathways were significantly increased during fermentation, including L-arginine, L-aspartic acid, leucine, L-lysine, citrate, and inosine (Figure 8, *P* < 0.05). Some products, such as carnitine, betaine, and thiamine, were only significantly increased in the fermentation broths under aerobic conditions (*P* < 0.05). This is consistent with a previous study on the fermentation process of shrimp sauce, during which the amino acid content increased continuously (21). Moreover, the content of arginine increased with the addition of lactic acid bacteria to the fermented dough (22). After Chinese wolfberry juice was fermented by lactic acid bacteria, betaine levels increased from 8237.77 to ca. 20,000 mg/L (23). During the fermentation process of peppers, the arginine content was higher in peppers inoculated with lactic acid bacteria than that of the control group without inoculation of lactic acid bacteria (24). Also, other studies (25, 26) also show that during *L. plantarum* fermentation, the amino acid content of the fermentation system was significantly increased (*p* < 0.05), which is consistent with the results of this study.

**FIGURE 9:**
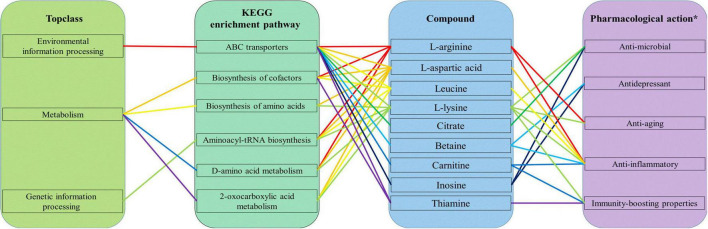
The top-class of the KEGG enrichment pathway and pharmacological action of nine main compounds produced by *Lactiplantibacillus plantarum* during fermentation of Polygonati Rhizoma; *information on the pharmacological action is obtained from the literature (21–38).

The nine main compounds enriched in these two pathways produced by *L. plantarum* during fermentation of Polygonati Rhizoma are also enriched in other KEGG pathways, including biosynthesis of cofactors, aminoacyl-tRNA biosynthesis, D-amino acid metabolism, 2-oxocarboxylic acid metabolism, in the top-class of environmental information processing, metabolism, and genetic information processing (Figure 9). These compounds are associated with a large number of pharmacological effects. For example, betaine can treat alcohol-related and metabolism-associated fatty liver disease and protect other tissues (27), while thiamine has the ability to regulate hyperglycemia and offers cardio protection (28). In addition, these compounds have been reported to have other properties such as anti-microbial (29–31), anti-depressant (32, 33), anti-aging (34, 35), anti-inflammatory (36–41), and immunity-boosting properties (42–44).

Furthermore, the amino acids, peptides, and analogs produced during these fermentation processes have a major impact on the enhancement of food flavor and functional effects. Aerobic conditions produce more flavor compounds like cinnamaldehyde and glutamate, etc. Cinnamaldehyde is fragrant with soap, wax, and violet flowers (45), and glutamic acid presents an umami flavor (46). These aroma components could enrich the flavor of fermented Polygonati Rhizoma. Peptide Telaprevir (VX-950) was also significantly increased under aerobic conditions, which is an inhibitor of the hepatitis C virus (HCV) NS3/4A protease (47). Functional substances such as isoleucine, L-alanine and L-homoserine are higher in the aerobic environment.

### 3.7. Regulation of amino acid biosynthesis

**FIGURE 10:**
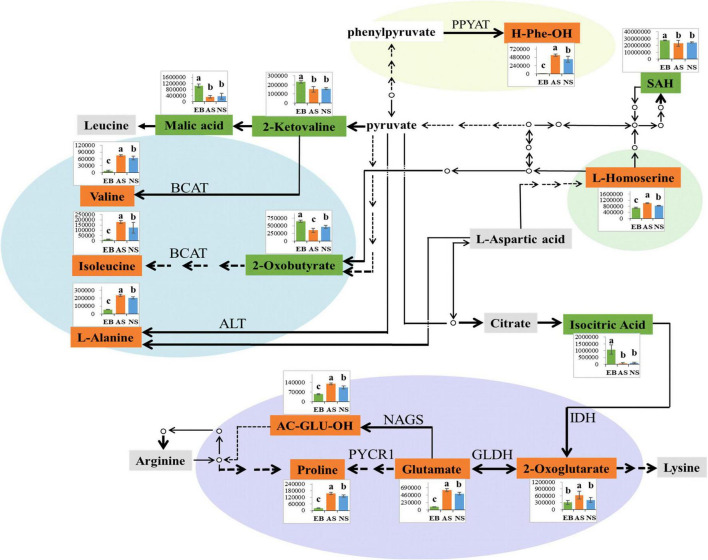
Metabolic pathway visualization and relative metabolite abundance of Polygonati Rhizoma fermented by *Lactiplantibacillus plantarum* under aerobic and anaerobic conditions. EB, extracts of Polygonati Rhizoma before fermentation; AS, aerobic fermentation supernatants; NS, anaerobic fermentation supernatants; significant differences were identified by the ANOVA test (*p* < 0.05); the compound displayed in green correlates with the highest content in EB; the compound displayed in orange correlates with the highest content in AS; the compound displayed in gray correlates with the highest content in both AS and NS compared with EB; PPYAT, phenylpyruvate aminotransferase; BCAT, branched-chain amino acid transaminase; NAGS, N-acetylglutamate synthase; IDH, isocitrate dehydrogenase; GLDH, glutamate dehydrogenase; PYCR1, Pyrroline-5-carboxylate reductase 1; H-Phe-OH, phenylalanine; SAH, S-Adenosyl-L-homocysteine; AC-GLU-OH, N-Acetyl-L-glutamate.

Interestingly, as shown in Figure 10, not only did the broths have varying amino acid contents before and after fermentation, but the amino acids in aerobic and anaerobic environments were also different. Compared with the anaerobic environment, the aerobic environment had significantly higher levels of L-alanine, valine, and isoleucine, all of which can be synthesized from pyruvate (*P* < 0.05). To be specific, L-alanine is synthesized from pyruvate through a synthesis catalyzed by alanine aminotransferase (ALT) (48). The synthesis of valine first generates 2-ketovaline from pyruvate through a series of reactions such as decarboxylation, condensation, reduction, replacement, dehydration, and further transamination to valine (48). In isoleucine synthesis, pyruvate is first converted to 2-oxobutyrate and then transaminated to isoleucine (48). During the synthesis of valine and isoleucine, branched-chain amino acid transaminase (BCAT) catalyzes transamination is regarded as the key enzyme in this reaction (49). From the findings of this study, it can be speculated that the activity of ALT and BCAT is enhanced under aerobic conditions, while their enzymatic activity is inhibited under anaerobic conditions. In a study on carp low-salt fermentation, the BCAT enzyme activity also showed similar results with the change in fermentation (50).

AS also had significantly higher levels of phenylalanine and L-homoserine than NS. It is speculated that the associated synthetase activity might be affected by the aerobic and anaerobic environments (*P* < 0.05). For example, phenylalanine can be synthesized by phenylpyruvate aminotransferase (PPYAT) catalyzed by phenylpyruvate (48). This could be attributed to the fact that the enzymatic activities of PPYAT are activated by oxygen under aerobic conditions, and vice versa. Previous study have demonstrated that the activity of PPYAT could be influenced by different substances; for example, its activity on *S. cerevisiae* was higher in cassava beer than in malt beer (51). However, there are few studies investigating the enzymatic activity of PPYAT in aerobic and anaerobic environments.

Glutamate, proline, N-Acetyl-L-glutamate, and 2-oxoglutarate also demonstrated similar trends. The current results are consistent with previous research findings, in which the amino acid content of tempeh increased significantly during aerobic fermentation but increased insignificantly during anaerobic fermentation (*P* < 0.05) (52). Glutamate can be synthesized using 2-oxoglutarate catalyzed by glutamate dehydrogenase (GLDH) (53). In the present study, AS had significantly higher levels of proline and N-acetylglutamic acid than NS. After 2-oxoglutarate generates glutamate, glutamate undergoes reduction, spontaneous cyclization, and finally catalyzed reduction to proline under the action of pyrroline-5-carboxylate reductase 1 (PYCR1). Several plant species tend to accumulate proline in response to environmental stress, and the upregulation of proline can eliminate reactive oxygen species and inhibit lipid peroxidation, thus enabling plants to adapt to various environmental stresses (54). N-acetylglutamate can be generated from glutamate through N-acetylglutamate synthase (NAGS) catalysis (55). Moreover, the content of 2-oxoglutarate in AS was also higher than that in NS, and 2-oxoglutarate can be catalyzed by isocitrate through isocitrate dehydrogenase (IDH) (*P* < 0.05). Therefore, it is postulated that the activities of IDH, GLDH, NAGS, and PYCR1 increase in an aerobic environment, but are inhibited by anaerobic conditions. Taken together, these findings indicate that the biosynthesis of *L. plantarum* metabolites is affected by aerobic and anaerobic environments, and this influence may play a crucial role in the further development of Polygonati Rhizoma products.

## 4. Conclusion

As a result of the fermentation process, certain bioactive metabolites were produced. The levels of organic acids, amino acids and peptides increased in both anaerobic and aerobic environments, and valine, isoleucine, L-alanine, proline, glutamate, 2-oxoglutarate, and L-homoserine, and other compounds are especially higher in aerobic environments. The continuous decline of pH value in the process of aerobic fermentation could be related to the increase of organic acids and amino acids. In summary, aerobic fermentation is more beneficial for the fermentation of Polygonati Rhizoma by *L. plantarum* to produce flavor and functional substances. Our findings provide insights that would be useful in the research and development of this medicinal and edible substance.

## Data availability statement

The original contributions presented in this study are included in this article/Supplementary material, further inquiries can be directed to the corresponding author.

## Author contributions

JJ: conceptualization and writing—review and editing. ZW: methodology. XK: bioreactor design. XL and CZ: data curation. ZW and ZX: writing—original draft preparation. JJ and SZ: supervision. JL: project administration. WH: funding acquisition. All authors have read and agreed to the published version of the manuscript.
