# Supplementary material for: Insights into the metabolic profiling of Polygonati Rhizoma fermented by Lactiplantibacillus plantarum under aerobic and anaerobic conditions using a UHPLC-QE-MS/MS system
Source: Front Nutr. 2023 Jan 26;10:1093761. doi: 10.3389/fnut.2023.1093761 (PMC9908587; doi:10.3389/fnut.2023.1093761)
Supplement: Supplementary file 1 [file Data_Sheet_1.PDF]

Supplementary material

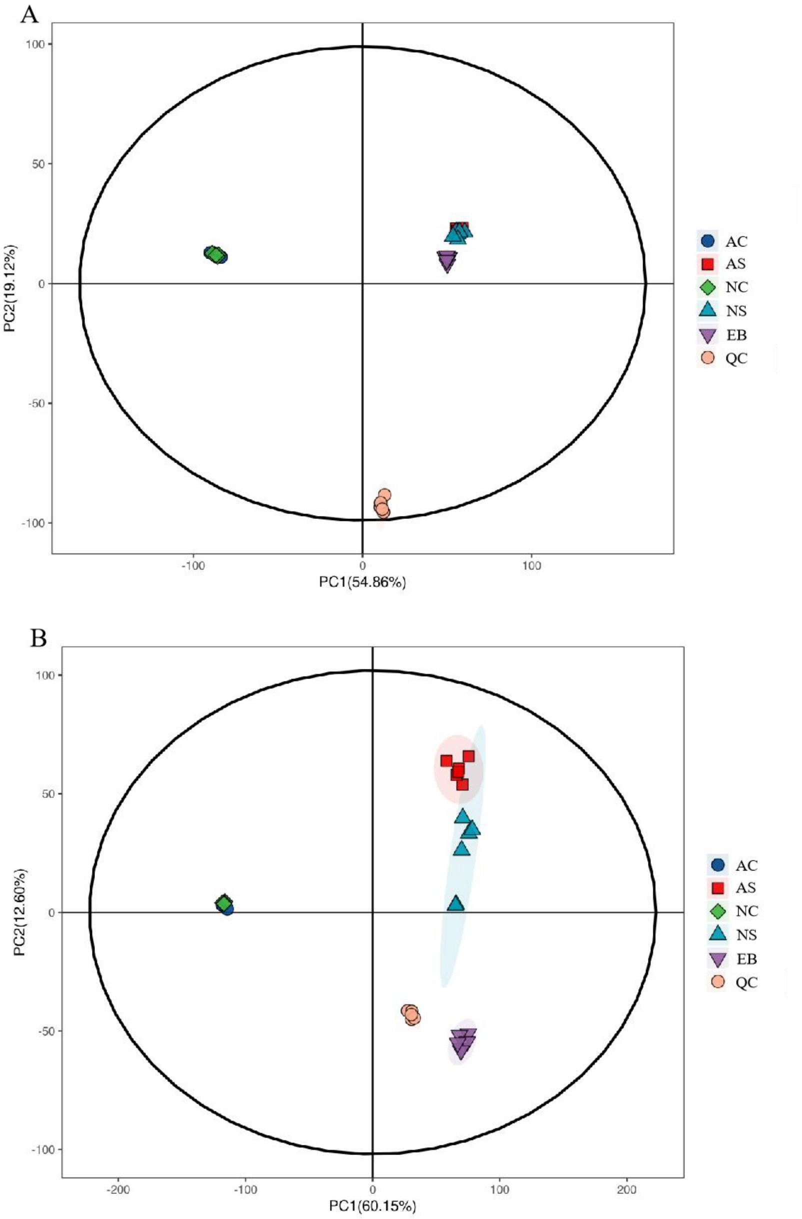

Fig. S1. PCA score plots of positive(A) and negative(B) metabolite profiling data of Polygonati Rhizoma fermented using *Lactobacillus plantarum* under aerobic and anaerobic conditions with QC samples. AC, aerobic fermentation *L. plantarum* cells; NC, anaerobic fermentation *L. plantarum* cells; AS, aerobic fermentation supernatants; NS, anaerobic fermentation supernatants; EB, extracts of Polygonati Rhizoma before fermentation; QC, Quality control.
